# Biodemographic perspectives for epidemiologists

**DOI:** 10.1186/1742-7622-2-10

**Published:** 2005-09-30

**Authors:** S Jay Olshansky, Mark Grant, Jacob Brody, Bruce A Carnes

**Affiliations:** 1 School of Public Health, University of Illinois at Chicago, Chicago, Illinois, USA; 2 Reynolds Department of Geriatric Medicine, University of Oklahoma Health Sciences Center, University of Oklahoma, Oklahoma City, Oklahoma, USA

## Abstract

A new scientific discipline arose in the late 20^th ^century known as biodemography. When applied to aging, biodemography is the scientific study of common age patterns and causes of death observed among humans and other sexually reproducing species and the biological forces that contribute to them. Biodemography is interdisciplinary, involving a combination of the population sciences and such fields as molecular and evolutionary biology. Researchers in this emerging field have discovered attributes of aging and death in humans that may very well change the way epidemiologists view and study the causes and expression of disease. In this paper, the biodemography of aging is introduced in light of traditional epidemiologic models of disease causation and death.

## Introduction

The timing with which death occurs and the underlying causes that contribute to it in humans and other sexually reproducing species have been the subject of scientific inquiry for hundreds of years, occupying the minds of scientists from widely ranging scientific disciplines [1]. For example, actuaries (who study humans exclusively) focus on the practical use of death statistics, as in calculating premiums for insurance companies or forecasting survival in order to assess the solvency of age-entitlement programs [2-4]. Demographers have historically worked in much the same way as actuaries, also on a single species, by explaining and understanding the trends, causes, and consequences of mortality within and between subgroups of the population and across time. In modern times, population biologists and entomologists have begun to study the demography of death among species other than humans [5-7]. Unlike demographers, epidemiologists invoke a disease-specific approach that has its historical roots in the study of infectious disease epidemics, with a subsequent focus on chronic diseases and conditions. In contrast to the scientists who work at the level of populations, biogerontologists examine death for humans and other species from a micro perspective as they endeavor to explain and understand changes that occur across time in molecules, cells, tissues, and organs that eventually contribute to disease and death.

The biodemography of aging is a "new" scientific discipline [7-9], arising in the late 20^th ^century as a product of efforts to merge the micro analysis of mortality explored by biologists with the macro analysis of scientists who do research at the population level (e.g., demographers and epidemiologists) in order to provide a biological rationale for the timing and causes of death in humans and other sexually reproducing species.

The earliest antecedents to the modern biodemography of aging date back to the French zoologist Georges Buffon, who demonstrated that "physical laws" regulate the duration of life in humans and other species [10]. These physical laws, according to Buffon, link the biological clocks that govern growth and development to similar clocks that he thought influenced duration of life. British actuary Benjamin Gompertz provided the first mathematical support for this view by demonstrating that for a significant portion of the human lifespan, the risk of death rises exponentially with age [11]. The linkage between Buffon's early life events and Gompertz's regularity in the timing of death appeared many years later with empirical evidence demonstrating that for some species duration of life is calibrated to the onset and length of the reproductive window [12]. These observations serve as the foundation for the biodemography of aging, and might profitably influence the traditional epidemiologic view of disease and death.

### An epidemiological view of disease and death

Epidemiology developed from the long history of human experience with infectious disease epidemics. An empirically-based science arising during the 18^th ^and 19^th ^centuries, the driving force of the discipline is an effort to explain and understand in humans, non-random health-related attributes of small and large populations, ranging from the clustering of fevers to age shifts in populations across time [13]. The necessity for more and better data led epidemiologists to forge collaborative ties with policy makers and biostatisticians in order to develop reliable reporting systems for vital events, such as births, deaths, and the identification and distribution of specific diseases. These collaborations with scientists from other disciplines exemplify the important interdisciplinary background of the field [14].

The biology of association (with the exception of genetic heterogeneity) is neither the main focus of epidemiology, nor does it assume great prominence in epidemiologic theory or methods. "Biological plausibility" is widely used in epidemiologic practice as a pragmatic gauge of the possible relevance of observed associations. Epidemiologists strive to understand causal factors (genetic or otherwise) that contribute to age-associated diseases. While epidemiologists have an appreciation of the importance of the aging process in disease expression, aging per se is not a focus of research in the discipline. As such, answers to questions about the lifespan of humans, or any other species for that matter, are outside the usual purview of epidemiology.

During the 20^th ^century, epidemiologists and others in the field of public health contributed to the first leap in human life expectancy, as infectious diseases yielded to improved sanitation, clean water, better diets, and increasingly more insulated living and working environments. Rapid declines in death rates at younger ages led to a redistribution of death from the young to the old, contributing to the accompanying 30-year rise in life expectancy that occurred over the century. Although the rise in life expectancy in the 20^th ^century is a triumph of public health and modern medicine, the price paid for this great success is a developed world dominated by the dual demographic landmarks of population growth and population aging [15], and a shift in underlying causes of death from infectious and parasitic diseases to chronic degenerative diseases expressed at middle and older ages [16]. The field of epidemiology naturally shifted some of its attention away from infectious diseases to the causation and prevention of chronic degenerative diseases.

Although scientific studies based on theoretical and methodological principles of epidemiology have been instrumental in identifying risk factors for chronic degenerative diseases (e.g., smoking, fatty foods, obesity, stress, etc., and their effects on heart disease, stroke, and many cancers) [17,18], the relative effects of these interventions on life expectancy at birth are much smaller than those that resulted from the control of infectious diseases early in life [19]. This occurs, in part, because the number of person-years-of-life (PYL) added to the life table when a child is saved from death is almost always considerably greater than the PYL added when an older person has their life extended. In addition, while preventive measures have been shown to reduce death rates at middle and older ages among population subgroups that adhere to healthy behavioral practices [20], most of the population has not adopted these practices. Diminishing increases in life expectancy at birth in developed nations today, despite the intensive efforts by epidemiologists and other public health experts to modulate lifestyles, present a paradox that can also benefit from a biodemographic examination of aging and disease.

### A biodemographic view of disease and death

Before examining epidemiology from a biodemographic perspective, it is important to understand the basic theoretical rationale supporting this new paradigm. The modern notion of the biodemography of aging arose in the early 1990s, as scientists from a broad range of scientific backgrounds began speculating about the biological forces responsible for the similar age patterns of death that they observed among sexually-reproducing species. The theoretical basis for the biodemography of aging is derived principally from evolutionary biology [21-24], but it has roots in historical efforts of scientists who speculated on what was referred to as a "law of mortality" [11,25-30] – an observation that a consistent age pattern of death is known to occur across species [12,31-34]. Modern biodemographers have theorized that sexually-reproducing species, including humans, experience a common age pattern of death because the aging of individuals, and by implication the aging of populations, is calibrated to life history traits whose own evolution was unrelated to either aging or duration of life – namely, the biology and timing of reproduction [8,12].

The link between death and reproduction is based on the evolutionary principle that the force of natural selection begins to decline rapidly once reproduction commences, approaching negligible levels at the end of the reproductive window (i.e., at menopause) [34]. The force of natural selection refers to the ability of selection to influence the distribution and frequency of alleles in the population – a force inherently linked to reproduction. As selection wanes, alleles with adverse health consequences expressed at progressively older ages can accumulate in the gene pool [21-24,35,36]. Evolution is blind to the health consequences of genes expressed in older regions of the lifespan because there is no selection to act upon them once they have been propagated. Empirical tests of this hypothesis have shown that the age trajectory of death is, in fact, a species-specific phenomenon that, as predicted from evolution theory, is calibrated to the onset and length of a species' reproductive window [12]. The reproductive window, in turn, is a genetically determined attribute that is established as part of a life history strategy that has been molded by the environment within which each species evolved. What this biodemographic perspective has taught us thus far is that for each species there is a fundamental link between the timing of sexual maturation, the length of the reproductive window, and the rate of increase in the death rate from biological causes of death. Thus, there is biology in the life table as originally anticipated by Benjamin Gompertz – a biology that Buffon [10] speculated on in the 18^th ^century, and which is driven by evolutionary forces that operate through genetic mechanisms.

Natural selection, the very heart of Darwin's theory of evolution, was based on the biological consequences of departures from a norm. Darwin's observations about imperfections in the morphological characteristics of living things were inconsistent with the works of an intelligent designer, which led him to the idea that all forms of life have an evolutionary history based on change over time. The life history details such as growth, development, and maturation that emerge from this unique evolutionary history of every species are central to understanding the variations that exist between individuals and species (including humans) in the temporal distribution of age-determined diseases and the timing of death [37]. Finally, the implications that the biodemography of aging has for the degree to which chronic degenerative diseases can be influenced by risk factor modification may be of even greater relevance to epidemiologists – the goal of chronic disease epidemiology.

An implied perfection of the human body is a persistent concept that emerged from the major World religions and has appeared repeatedly in legends from almost every culture dating back to antiquity [38]. These images of perfection describe a distant past when humans were either immortal or extremely long-lived. The most common historical explanation for the loss of immortality, the lack of perfect health, and the steady decline in human longevity has been that each new generation has adopted increasingly more decadent lifestyles. Roger Bacon, an influential English philosopher and scientist of the 13^th ^century, was the first to popularize this view [39]. However, he also believed that the trend toward shorter lifespans could be reversed by invoking the "secret arts" of the past – namely, the adoption of more austere lifestyles and the ingestion of foods and other substances believed to have life-extending properties. Thus, the perspective that aging and diseases are amenable to modification through changes in lifestyles, has its origins in thinking that extends back in time at least one thousand years.

This persistent belief in perfection and the consequences of a departure from it has spawned two other beliefs that continue to have a significant philosophical and practical influence on contemporary scientific views of mortality. The most important of these is the belief that aging and diseases are unnatural and are, therefore, somehow avoidable. The second is the notion that the health and longevity consequences associated with perfection can be reclaimed through human actions. Elements of this latter idea contribute to modern epidemiologic thought. These beliefs, and the quest for longer lives that arises from them, have been obsessions throughout human history [40], having become a central part of the paradigm of modern medicine and the effort of epidemiologists to understand how risk factors alter death rates.

In modern times, aging is described by some as a disease that can be reversed, slowed, or even eliminated by changes in lifestyles or by ingesting vitamins, minerals, anti-oxidants, and hormones – modern versions of the anti-aging remedies of the past [41,42]. On the surface, this philosophy of personal empowerment is seductive. However, a biodemographic perspective, which is based in part on an examination of the anatomical structures and functions of the human body, raises doubts about the validity of this perspective. For example, from a biodemographic perspective it is suggested that aging and many of the diseases that accompany it are not deviant departures from perfection, or even the sole consequence of moderately decadent lifestyles. Instead, they are primarily the consequence of operating our bodies beyond their biological warranty period [43] (i.e., beyond the time when parents can contribute to the reproductive fitness of their own offspring). Thus, the philosophy that people are empowered to control their own disease, aging, and longevity has become, in modern times, an ideology of personal blame. In effect, it has become common to blame people for many of the diseases and disorders that they experience as they age, and more importantly, some may be led to believe that aging and the diseases that accompany it are largely avoidable. From a biodemographic perspective, it is certainly true that some diseases and disorders are entirely preventable and that aging and death can be hastened by imprudent lifestyles, but once these harmful lifestyles are avoided, most of what is commonly recognized as aging and disease is an inevitable by-product of operating the machinery of life. Even though aging, disease, and death are not programmed into our genes, once the engine of life switches on, aging is inevitable.

Herein lies the link between epidemiology and biodemography. Modern epidemiology arose out of an infectious disease paradigm where treatments and prevention were shown to dramatically reduce the incidence and prevalence of communicable diseases. Once the epidemiologic transition from high to low mortality permitted most people to survive beyond the end of their reproductive window, chronic degenerative diseases appeared with rising frequency. Similar epidemiologic approaches to disease modification were then applied to these diseases expressed mostly at middle and older ages. Although the results of this effort have often been dramatically successful (e.g., established linkages between smoking and cancer; obesity and diabetes; and high blood pressure and stroke), the resulting behavioral modifications have not led to another quantum leap in life expectancy like that observed during the 20^th ^century. Instead, what occurred was a steady decline in death rates accompanied by a diminishing rise in life expectancy at birth and at older ages. The phenomenon of diminishing longevity gains from lifestyle modification is referred to here as the *Medawarian Paradox*, named after Sir Peter Medawar [23] who suggested that aging is "...revealed and made manifest only by the most unnatural experiment of prolonging an animal's life by sheltering it from the hazards of its ordinary existence" (p.13).

The dramatic increase in the last century in the number of people living for seven decades or more [44] has revealed an entirely new set of "weak links" in the structure and function of the human body that are associated with living well beyond our reproductive years [45,46]. Extended survival into the post-reproductive period permits a number of anatomical and physiological features of the human body to reveal themselves as debilitating diseases and disorders such as Alzheimer's disease and osteoporosis. These weak links were not commonly known or thought of as such in the past because they were uncommon – people rarely lived long enough to experience them. We define them as "weak links" now because of the Medawarian Paradox of the unusual circumstance of survival into older ages.

It is important to emphasize that the concept of anatomical oddities and weak links in humans and other living things is not new. The idea began when Charles Darwin suggested that imperfections in the design and functioning of parts of living things are inevitable by-products of natural selection's blind eye to body design. According to evolution theory, selection does not operate with any particular goal in mind; it simply optimizes the perpetuation of DNA across time by constructing bodies capable of carrying the DNA and passing it successfully from one generation to the next. This idea has since appeared several times in the published literature in the 20^th ^century, including the first detailed presentation of morphology and body design [47], in later publications by evolutionary theorists [48-51], and in modern discussion of reliability theory [33]. However, neither Darwin nor those who followed in his footsteps ever examined the morphology of living things from the perspective of an aging animal.

### Epidemiology of degenerative diseases in an aging world

The theoretical and methodological basis for epidemiology arose out of a communicable disease model where a notion of avoiding and curing the common fatal diseases of the time was prevalent and enormously successful [35]. As a measure of that success, half of the gain in life expectancy at birth in the 20^th ^century was achieved by 1920 – largely as a result of public health interventions. As life expectancy increased, chronic diseases became the overwhelming cause of death, with diseases of the heart, cancer, and cerebrovascular disease accounting for 70 percent of all mortality. These diseases do not have a single cause, but usually result from a complex web of causation. The concept of risk factors was introduced to facilitate understanding and prevention. Thus, smoking was shown to increase the risk of heart disease and cancers. Fatty foods, obesity, and lack of exercise clearly predisposed people to these conditions [52]. However, frustration arose over the years, as it was shown that these risk factors accounted at best for only half of the deaths from these three causes. It is now realized that such phenomena as environment, psychosocial factors, and genetics also play important roles [53].

In general, control of chronic diseases has been effective enough to produce a gradual increase in life expectancy, albeit at a diminishing rate. In developed countries, where life expectancy is about 75 years for males and 80 years for females, the classic indicators of health are becoming less useful. Infant mortality has been very low for more than 20 years and the concept of "premature death" is no longer a sensitive health indicator [54]. Instead, with each succeeding year lived at the tip of the exponential curve of mortality, numerous detrimental physiologic mechanisms and morbidities are activated. Although one role of the epidemiologist is to improve quality of life, often at best what can be accomplished is slowing the progression of diseases and conditions expressed in older regions of the lifespan by suppressing their symptoms. These include Alzheimer's disease and related dementias, vision and hearing loss, hip fracture, osteoporosis and osteoarthritis, incontinence, depression, social isolation, widowhood, and institutionalization [55]. *In an aging world where the envelope of human survival is continuing to be extended, a biodemographic perspective leads to the realization that the expression of disease becomes more a by-product of extended survival rather than the end product of identifiable and modifiable risk factors.*

## Conclusion

When the focus of epidemiology shifted from communicable diseases to chronic degenerative diseases, the process of aging intruded into the traditional epidemiologic model that links behaviors to mortality risks and life expectancy determination. Now that human survival has been progressively extended deeper into the post-reproductive period of the lifespan, a biodemographic perspective reveals biochemical and biomechanical forces that have a profound influence on population frailty, disease expression, and duration of life. Biochemical constraints involve the inevitable accumulation of damage that occurs at all levels of biological organization, including the maintenance and repair processes themselves – a loss of biological fidelity that most biogerontologists suggest cannot as yet be modulated [44,56]. Biomechanical forces involve the progressive and currently immutable diminishment of structure and function in the very morphological features that give species their phylogenetic identity [40,46,49,50]. Anticipated advances in research (e.g., caloric restriction mimetics, embryonic stem cells, and progress in the replacement of body parts) will continue to erode the biochemical and biomechanical barriers to longevity and quality of life.

An important message in this paper is that biology reminds us that evolutionary success does not require living to old age, it only requires living long enough to reproduce. Our bodies fail over time not because they were designed to fall victim to aging and disease at a predetermined age [57], or even because of the acquisition of risk factors and decadent lifestyles, but because they were not designed for extended operation. The diseases and disorders we experience in the post-reproductive period of the lifespan are, therefore, not flaws from an evolutionary perspective, and unless proven otherwise, should not be attributed to personal failure or exclusive by-products of environmental risk factors. The biological consequences of aging are crucial factors for epidemiologists, whose concepts and methods for the pursuit of specific causes and risk factors are not entirely applicable to animals living long enough to experience the Medawarian Paradox.

Those of us alive today are the most recent recipients of an evolutionary legacy that includes a human body filled with both awe-inspiring complexity, and a host of anatomical oddities and weak links that are revealed with the passage of time. The diseases and disorders that arise from the negative side of this legacy are unfortunate and unanticipated by-products of the human ingenuity that has allowed our bodies to be operated far longer than nature has historically permitted. Had natural selection operated with a particular goal in mind, such as a healthy old age, there is reason to believe that the morphological structures and biochemical makeup of sexually-reproducing species would probably be far different from what is currently the case [46]. It is important to remember that in an aging world the expression of disease and how long we live as both individuals and populations is more a product of evolutionary neglect, not evolutionary intent. As such, traditional epidemiologic models should become more sensitive to the unique biological forces that come into play in a world where aging is common and biochemical and biomechanical forces have an important influence on disease expression and length of life.

## Competing interests

The author(s) declare that they have no competing interests.
